# Ischemic cardiomyopathy revealed by central retinal artery occlusion (CRAO)

**DOI:** 10.11604/pamj.2015.22.250.7308

**Published:** 2015-11-17

**Authors:** Ihsen Zairi, Khadija Mzoughi, Zouhayer Jnifene, Fethia Ben Moussa, Sofiene Kammoun, Sana Fennira, Nidhal Ben Moussa, Jihen Brour, Monia Cheour, Sondos Kraiem

**Affiliations:** 1 Department of Cardiology, Habib Thameur public hospital, Bab el Fallah, Tunis, Tunisia; 2 Department of Ophthalmology, Habib Thameur public hospital, Bab el Fallah, Tunis, Tunisia

**Keywords:** Central retinal artery occlusion, ischemic cardiomyopathy, cardiac thrombus

## Abstract

Here we report a case of central retinal artery occlusionrevealing an ischemic cardiomyopathy. A 54-year old smoker man presented at the hospital because of sudden visual loss in his left eye. There was cherry-red spot in the macula in his left eye. We performed a fluorescein angiogram and cervical color Doppler. Later investigations revealed an ischemic cardiomyopathy undiagnosed until then.

## Introduction

Central retinal artery occlusion is a rare but one of the ophthalmological emergency with worse prognosis that causes definite loss of vision within few hours of installation. Assimilated to the ocular analogue of cerebral stroke, it results on impaired functional capacity and quality of life. After initial measures, further investigations are necessaryand can suggest the etiology of the CRAO.

## Patient and observation

**Figure 1:**
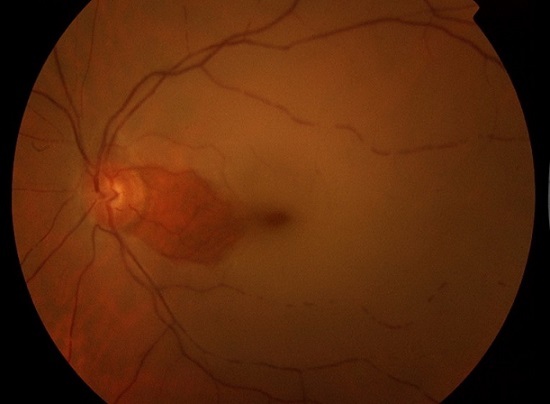
Colour fundus photograph of the left eye showing acute CRAO with cherry-red spot and cattle trucking of the arterioles

**Figure 2:**
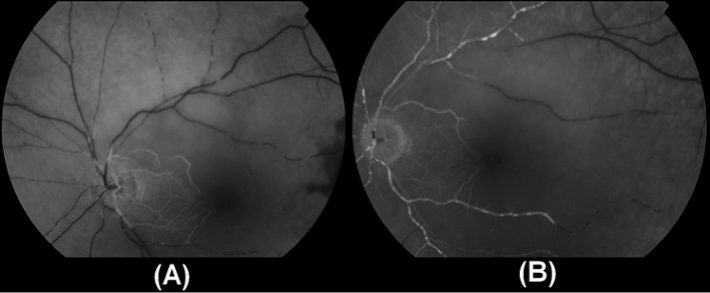
Fundus fluoresce in angiography. (A) 25seconds from injection: no distal opacification; (B) 180seconds from injection: multiple intra-arterial emboli with delayed opacification of distal branches

A 54-year-oldmanwasadmittedtoHabib Thameur HospitalbecauseSudden loss of vision in the left eyefrom 12 hours. He is a smoker with nopastmedicalhistory. Cardiovascular exam revealed no abnormalities. Color fundus photograph of the left eye showing acute CRAO with cherry-red spot and cattle trucking of the arteriole (Figure 1). Fundus fluorescein angiography showed adelayedarterial transit time (Figure 2). After 25seconds from injection, there was no distal opacification. 180seconds later, multiple intra-arterial emboli were noticed.Ophthalmologist concluded to a central retinal artery occlusion. After initial management, the patient was referred to cardiology department for further investigations. He revealed a typical stable angina from two months. The EKG showed Antero-septal necrosis (QS from V1 to V3).

Trans-thoracicechocardiogram exam (apical four-chamber view) demonstrated a giant thrombus attached at the left ventricular apex, an impaired left ventricular ejection fraction with an antero-septal akinesis. Supra Aortic Doppler showed no stenosis. Coronary angiography revealed right coronary artery occlusion and a left coronary artery dissection. Patient was referred for a coronary artery bypass graft surgery.Thepostoperative coursewas uneventful and the patient was discharged few days later.

## Discussion

Central retinal artery occlusion is a rare but one of the ophthalmological emergency with worse prognosis that causes definite loss of vision within few hours of installation [1]. The incidence is estimated to be 1 in 100 000 people and accounts for 1 in 10 000 ophthalmological outpatient visits [2]. Assimilated to the ocular analogue of cerebral stroke, it results on impaired functional capacity and quality of life. Central retinal artery occlusionsignifies end-organ ischemia and has the same risk factors that in turn place an individual at risk of future cerebral stroke and ischemic heart disease. Blood supply to the retina originates from the ophthalmic artery.

Typical funduscopic findings of a pale retina with a cherry red macula result from obstruction of blood flow to the retina from the retinal artery, causing pallor, and continued supply of blood to the choroid from the ciliary artery, resulting in a bright red coloration at the thinnest part of the retina. These findings do not develop until an hour or more after embolism, and they resolve within days of the acute event. By this time, visual loss is permanent and primary optic atrophy has developed. In those with a cilioretinal artery supplying the macula, a cherry red spot is not observed.

There is currently no guideline-endorsed evidence for treatment. Therapy optionsinclude sublingual isosorbidedinitrate, systemic pentoxifylline or inhalation of a carbogen, hyperbaric oxygen, ocular massage, globe compression, intravenous acetazolamide and mannitol, anterior chamber paracentesis, and methylprednisolone with no superiority when compared to placebo [3]. There has been recent interest in the use of tissue plasminogen activator (tPA) on the treatment of acute central retinal artery occlusion [4].

Causes of central retinal artery occlusion vary depending on the age and the riosk factors of the patient. An embolism, atherosclerotic changes, inflammatory endarteritis, angiospasm, or hydrostatic arterial occlusion may occlude the retinal artery. The mechanism of obstruction may be obvious from comorbid systemic disease or physical findings. Atrial fibrillation and ipsilateral carotid stenosis are more commonly associated with prolonged visual disturbances. Varma and al suggested a Vascular Workup for Patients withcentral retinal artery occlusion (Table 1) [1]. 64% of patients suffering a CRAO had at least one new undiagnosed vascular risk factor, the most common being hyperlipidaemia (36%), followed by hypertension (27%) and diabetes (12%) [5]. In addition, 27% of patients had an ipsilateral carotid stenosis of >50%. The result is analogous to another study, which found that 31% of patients had ipsilateral carotid stenosis of >50 and 71% had atherosclerotic plaques. In all, 52% had an abnormal echocardiogram, suggesting a cardioembolic source [6].

**Table 1:** Suggested vascular workup for patients with central retinal artery occlusion [1]

Common vascular risk factors (all patients)	Blood pressure
	Fasting lipids and lipid profile
	Fastingbloodsugar
Exclusion of arteritic CRAO Investigations for embolic sources	ESR, CRP, Platelet count
	Duplex carotidultrasound
	Echocardiogram
Younger patients (<50 years old) with no vascular risk factors	Thrombophilia screen (protein C and S, factor V Leiden, antiphospholipid antibody), vasculitic screen (ANA, ENA, ANCA, ACE)

Erythrocyte sedimentation rate (ESR), C-reactive protein (CRP)

In our case, the etiology was an Embolus from the heart is the most common cause of CRAO in patients younger than 40 years.Emboli to the retinal circulation may originate at any point in the proximal circulation from the heart to the ophthalmic artery [7].

## Conclusion

Ischemic cardiomyopathy is not rare and has the same risk factors that causes systemic vascular disease. Investigating by a cardiovascular examination should be one of the major steps in the Workup for Patients with central retinal artery occlusion.
